# Expressions of IL4, IL10, and IFNγ cytokines genes during bacterial mastitis

**DOI:** 10.5455/javar.2022.i567

**Published:** 2022-01-15

**Authors:** Rana A. Faaz, Fawziah A. Abdullah

**Affiliations:** Department of Microbiology, College of Veterinary Medicine, University of Basrah, Basrah, Iraq

**Keywords:** Cow mastitis, gene expression, inflammatory cytokine, somatic cells, *16S rRNA* gene

## Abstract

**Objective:**

Many bacteria are involved in causing mastitis in dairy cows. Perfect identification of bacteria is crucial for the appropriate choice of drug for treatment. This study aims to find out the various bacteria that cause mastitis through the 16S ribosomal ribonucleic acid (*16S rRNA*) gene.

**Materials and Methods:**

A total of 150 mastitis somatic cell samples were tested with bacterial nested polymerase chain reaction (PCR) universal primers, targeting the *16S rRNA* gene. The primers had both Gram-positive and Gram-negative bacterial specificities. Inflammatory cytokine interleukin (IL-10), IL-4, and interferon-gamma (IFNγ) expression genes were measured and compared in mastitis-free and mastitis-affected animals.

**Results:**

Based on the PCR, 70 (46.7%) samples showed positive results. The expression of the IL-10 gene was significantly higher (*p* < 0.001) in mastitis-affected cows than noninfected animals. Compared to cows diagnosed with clinical mastitis, the IL-4 and IFNγ genes were expressed more strongly in healthy cows (*p*> < 0.0001).

**Conclusion:**

Mastitis has been linked to both Gram-positive and Gram-negative bacteria. These genes are strong predictors of mastitis in the states analyzed, as evidenced by the differential expression in mastitis and healthy conditions of the IL-4, IL-10, and IFNγ genes. The genes examined here and others will be the subject of additional research.

## Introduction

Mastitis is the most common, costly, complex, and multifactorial dairy cow disease [1]. It is an infection or injury to the udder tissue in the mammary gland (MG) that causes inflammation in the MG. The etiopathology of cattle mastitis is multifaceted; it affects the udders of dairy cows and induces milk and mammary alterations. Due to decreased milk supply and low quality, it is the most common disease to cause a financial loss in the dairy sector. Mammary tissue damage accounts for 70% of the total loss, resulting in lower milk production [2].

The cow’s milk appears to be watery with flakes and clots. Peripartum and postpartum mastitis are two types of clinical mastitis. It can also include minor discomfort and abnormal milk that lasts longer than 2 months and is chronic. On the other hand, in addition to an inflammatory response that causes abnormal milk to appear, the modifications in the udder may include swelling, warmth, discomfort, and redness in the event of mild or mild clinical mastitis, which is referred to as extre [3].

Chronic mastitis is a long-term inflammatory illness with clinical flare-ups at irregular intervals [4]. Sub-clinical mastitis, unlike clinical mastitis, does not cause apparent abnormalities in the udder or milk, although milk output declines as the somatic cell count rise (SCC); clinical mastitis can be lethal in difficult situations. Although it is impossible to quantify the financial loss caused by sub-clinical mastitis, experts believe it costs the herd more money than clinical cases.

Intramammary infections most commonly cause mastitis. Several risk factors have been linked to the occurrence of bovine mastitis, including pathogen, host, and environmental factors. The mastitis control programs consider all of these issues; chemicals, physical, or pathological factors can induce mastitis, and changes can occur in glandular tissues [5].

Given the differences in the management practices used in various countries, different bacteria species have been associated with mastitis in other geographic locations [6]. Al-Haddadi et al. [7] found that environmental bacteria are the most often isolated bacteria, instead of the mild and infectious bacteria from clinical and subclinical mastitis. Many authors proved that environmental mastitis was caused by environmental pathogens and germs from the encircling surroundings, while contagious mastitis is handed down from other affected quarters. Communicating with hosts online on this mucosal surface is part of the primary line of innate defense [8]. Mammary epithelial cells (MEC) are excellent models for studying the host immune response in bacteria-precipitated mastitis [9].

As a result, determining corrective and preventive treatments for pathogen-induced mastitis in dairy cows may require a thorough understanding of the pathogen-specific molecular pathways implicated in the production of inflammatory retorts in the MG [10].

To colonize the udder, harmful bacteria enter the udder lumen through the teat canal. This colony development is thought to be a selective advantage for infections that cause mastitis, allowing bacteria to remain in the udder longer. Immune cells and chemoattractants are released when a pathogen interacts with bovine mammary epithelial (BME) cells, attracting and activating immune cells; localized antimicrobial activity properties are then used to intensify the inflammatory process [11].

As a result, BME cells are assumed to be at the front of the MGs’ bacterial infection resistance. In multiple studies, BME cells have been demonstrated to detect bacteria or biological chemicals and react slightly faster than genes linked to inflammatory reactions [12]. When produced in BME cells, toll-like receptors (TLRs) identify microbe-associated molecular patterns (MAMPs) and turn on the innate immune system of the MG. When MAMPs stimulate TLRs, a cascade of cellular processing events occurs, resulting in the release of cytokines, growth factors, and inflammasomes [13].

Pattern-recognition receptors (PRRs), which are germline-encoded and identify pathogen-associated molecular patterns, are required to activate innate immune responses such as infection [pathogen-associated molecular patterns (PAMPs)]. PRRs activate a variety of signaling events in response to the discovery of PAMPs. It has been proved by a number of research groups that these cells release chemoattractants that attract polymorphonuclear neutrophil leukocytes to the site of contamination, increasing the SCC, which indicates the different types of cells found in milk, such as leukocytes and epithelial cells. The significant rise in milk SCC during infection was mostly due to the influx of neutrophils from the bloodstream into the MG, with neutrophils accounting for over 90% of the leukocyte population in milk from infected udder quarters, compared to low levels in uninfected ones. The milk SCC is a diagnostic technique for sub-clinical mastitis sub-clinical mastitis (SCM) that detects the concentration of somatic cells, primarily inflammatory ones. A healthy udder’s SCCs were around 70,000 cells/ml, dependent on the cow’s age, breed, lactation stage, and milk yield. Using a threshold of 200,000 cells/ml, SCM can be discriminated against normal udders. However, some research have used a threshold of only 100,000 cells/ml. However, it has been proven that mammary inflammation can be generated [14].

Large-scale metagenomic investigations of those cell reactions to TLR stimulation are rare. It was mentioned that developing effective vaccines is complicated due to the excessive variety of mastitis diseases. Bulk tank milk sample testing is a precise and practical method for assessing milk quality at the herd level and is especially valuable for detecting and identifying infectious bacteria in cows with mastitis. To solve these problems and find suitable solutions, research should be conducted to understand the natural pathways regarding disease tolerance and identification. Mastitis protection is a complicated approach to addressing such issues. This research project aims to identify the bacterial pathogens responsible for bovine mastitis using nested polymerase chain reaction (PCR) and 16S-based phylogenetic analysis and to describe the immune system response gene expression pathways to mastitis using real-time PCR. Interleukin-4 (IL-4), IL-10, and interferon-gamma (IFNγ) genetic traits will be studied. 

## Materials and Methods

### Approval based on moral principles

Authorization No. 5593VMB for approval was obtained in February 2019. Samples were collected with the approval of the college, as well as the field owners. As for the laboratory work, it was carried out in the laboratories of the Faculty of Veterinary Medicine, University of Basra.

Between February and May of this year, researchers in the Iraqi province of Basrah collected milk samples from 70 cows that had been diagnosed with clinical mastitis. All the samples were sent for PCR investigation. Two groups of samples, one without infection and one with clinical mastitis, were selected to investigate the gene expression related to infections of the immune system response pathways. Each group comprised 10 animals. One-quarter of each cow’s milk was collected in 15-ml dilutions placed in centrifuge tubes. Before collecting the samples, all animals had their udders clinically examined.

### Isolation of somatic cells in milk

Aseptically, 2 ml of milk was collected and tested. Discarded test tube supernatant included lipids and soymilk. Tube 2 supernatant was centrifuged at 16,000 *g* for 5 min at 4°C, and repeated thrice. The remaining pellet in tube 1 was thoroughly cleaned with phosphate buffer saline (PBS) before being resuspended in 250 μl of phosphate buffer saline (PBF) solution (PBS). The supernatant of tube 2 was also discarded because it contained fat, whey, lipids, and soy milk. This was repeated thrice. The residual pellet in the test tubes was mixed thoroughly in 450 μl of PBF solution after being treated twice in PBS [15]. After that, the samples were used for deoxyribonucleic acid (DNA) and ribonucleic acid (RNA) extraction.

### Nested PCR 

Gene Aid DNA Extraction Kit (Korea) was used to recover genomic DNA from milk somatic cells. The extracted DNA was quantified on a 1% agarose gel stained with ethidium bromide using a Nanodrop spectrophotometer (Quawell, USA) at 260/280 nm. Subsequently, two universal primers with strong specificity for Gram-positive and Gram-negative bacteria were used to amplify the 16S ribosomal deoxyribonucleic acid(rDNA) gene fragment. The primers were used in the following order: outer forward UNI_OL 5′- GTG TAG CGG TGA AAT GCG-3′, outer reverse UNI_OR 5′-ACG GGC GGT GTG TAC AA-3′, inner forward UNI_IL 5′-GGT GGA GCA TGT GGT TTA-3′, inner reverse UNI_IR 5′-CCA TTG TAG CAC GTG TGT-3′. The PCR primers were designed and provided by Bioneer Company (Korea). The combination for the amplification constituted 29 μl of nuclease-free water, while each primer was 3 μl. 15 μl of template DNA PCR was processed (Techne, UK). The operation was conducted at 94°C for 5 min and then at 94°C for 1 min. External primer pair annealing took place at 55°C for 1 min, and elongation took 1 min at 72°C. The loop was carried out 33 times in total. In the second part of the nested PCR, one from the first run was used in the second part of the nested PCR. Under a UV-transilluminator, the external primer pair had a 709-bp amplicon. After gel electrophoresis in a 2% agarose gel, the internal primer pair had a 287-bp amplicon (Vilber Lourmal CE; Taiwan) with safety dye (Green DNA DYE, Biotech, USA) after gel electrophoresis. DNA was assessed on an agarose gel and quantified using a BioPhotometer plus (NanoVue, USA).

### DNA sequencing, sequence alignment, and phylogenetic analysis

The same primers used in PCR were used to sequence the 16S ribosomal ribonucleic acid (*16S rRNA*) gene fragments. PCR amplicons of bacterial 16S rDNA were sent to Macrogen Company Laboratory in Korea. The percentages of similarities and differences were calculated by visually inspecting the sequence alignments.

The alignments of the currently known bacteria’s sequences were compared with those of previously published bacterial organisms. For each strain of bacteria, *16S rRNA* PCR results were compared to those previously published (http://blast.ncbi.nlm.nih.gov/) based on the GenBank database’s extremely comparable sequences. Unweighted pair group method with arithmetic was used to investigate the evolutionary history. The tree was designed in dimensions, with branch lengths in the same units. The generational differences were improving, suggesting that the phylogenetic analysis was correct. The total cumulative probability method was used to measure the distances, which were measured throughout in terms of the number of reshaped for every location.

### Extraction of RNA and reverse transcription

A TRIzolTM Plus RNA Purification Kit (USA) was used as directed by the manufacturer to extract nucleic acid RNA. Milk somatic cell pellet was lysed with one milliliter of TRIzol reagent (1.0 mM). The mixture was centrifuged after being added to 0.2 ml of chloroform (phase separation). In the aqueous phase, the RNA was precipitated by combining it with an equal amount of isopropyl alcohol and washing it with 75% alcohol several times, followed by an appropriate volume of diethylpyrocarbonate-treated water. Using a BioPhotometer plus, the extracted RNA was spectrophotometrically quantified (NanoVue, USA). The OD260/OD280 ratio was used to assess efficiency. The reverse transcription reaction was started by mixing 1 μg of total RNA with 1 μg of (dT12-18) oligo in 12 μl of sterile, filtered water and heating it for 10 min to 70°C (232.2°F). After the mixture was refrigerated on ice, we added 0.5 mM of each of the dNTPs, 5 μl of first-strand buffer, and 200 U of SuperScript-II RNase-H Reverse Transcriptase. The mixture was prepared ahead of time by stabilizing it for 10 min at 25°C.

### Quantitative PCR

The manufacturer’s recommendations for quantitative PCR were followed. A SYBR green dye-based Master Mix Kit (Promega GoTaqTM qPCR, USA) was used. The primers used to assess gene expression were constructed according to published guidelines (Table 1). The Glyceraldehyde-3-phosphate dehydrogenase (GAPDH) gene was used as an endogenous guide. The reaction was in triplicate, and the reaction volume was set at 20 μl per sample, 5 μl of sample complemented deoxyribonucleic acid (cDNA), 1 μl of each forward and reverse primers and GAPDH and 13 μl sterile deionized water. To obtain the desired level of amplification, the reaction was heated to 95°C for 2 min, followed by 40 cycles of 30 sec at 95°C, 1 min at 55°C, and 1 min at 72°C The 2 Ct technique was used to do relative quantification on all of the samples, which were run in triplicate.

**Table 1.** Primer pairs used in quantitative real-time PCR to determine bovine cytokine gene expression.

Gene	Oligonucleotides (5’-3’) F: forward; R: reverse	Reference
*IL-4*	F: CATGCATGGAGCTGCCTGTA R: AATTCCAACCCTGCAGAAGGT	[16]
*IL-10*	F: CCAAGCCTTGTCGGAAATGA R: GTTCACGTGCTCCTTGATGTCA	[17]
*IFNγ*	F: TGGATATCATCAAGCAAGACATGTT R: ACGTCATTCATCACTTTCATGAGTTC	[18]
*GAPDH*	F: GGCGTGAACCACGAGAAGTATAA R: CCCTCCACGATGCCAAAGT	[18]

**Figure 1.**
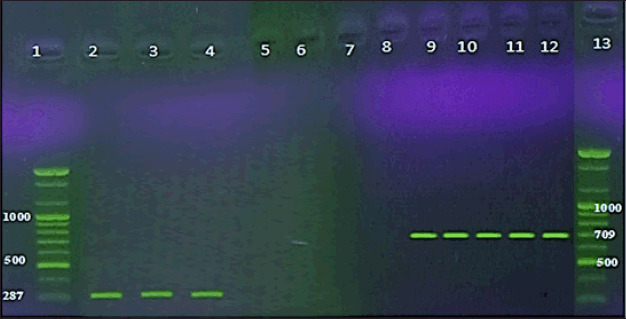
Nested PCR detection of DNA from the somatic cell of cow mastitis. Lanes 1 and 13, DNA marker. Lanes 2–4 (287-bp). Lanes 8–12 (709-bp).

### Statistical analysis

The *t*-test was conducted with a 5% significance threshold to see any correlation between the results. SPSS software version 22 is used for statistical analysis.

## Results

### Nested PCR

DNA from mastitis was predicted to be multiplied by uniform bacterial sections of the *16S rRNA* gene. Milk somatic cells using UNI OL, UNI OR, UNI IL, and UNI IR were also primers. The planned nested PCR primer pairs were tested. The 150-mastitis milk samples’ somatic cells and the *16S rRNA* were amplified in 70 (46.7%) of the samples tested. In 70 (46.7%) cases, amplicons of projected wavelengths (709 and 287-bp) were observed with the first and second nested PCR runs, referring to *Staphylococcus aureus, S. epidermidis*, *Streptococcus pyogenes*, *S. agalactiae*,* Enterococcus faecalis*,* Klebsiella pneumoniae*,* Serratia marcescens*,and* Pseudomonas aeruginosa* (Fig. 1).

### DNA sequences and sequence alignment of 16S rRNA 

**Figure 2.**
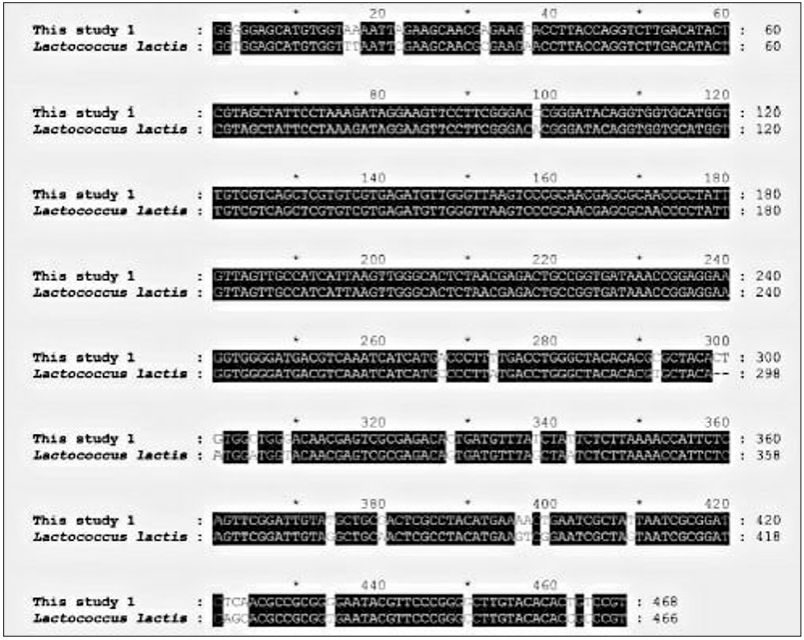
Alignment of *16s rRNA* nucleotide sequences of *L. lactis. L. lactis* (MT305928.1) represents the reference sequence obtained from the National Center for Biotechnology Information (NCBI) website.

**Figure 3.**
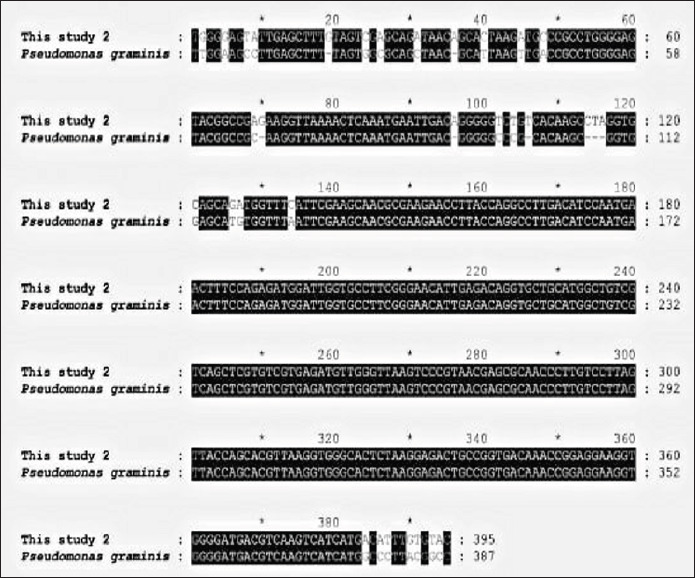
Alignment of *16s rRNA*nucleotide sequences of *P. graminis. P. graminis*(KU523561.1) represents the reference sequence obtained from the NCBI website.

Sequencing and basic local alignment search tool comparative analyses confirmed the PCR product diagnosis even further. There were 93.38% sequence identities found between the published sequences of *Lactococcus lactis* (MT305928.1), which cover 100% of the gene bank, and the *16S rRNA* sequences from *P. graminis* (KU523561), which cover 94% of the gene bank and roughly 395 (Figs. 2and 3). This sequence was designated as *Lactococcus* sp. clone UOBVM and *Pseudomonas* sp. clone UOBVM, which were entered into the GenBank database with accession numbers MW020033 and MW020096, respectively.

### Phylogenetic analysis

**Figure 4.**
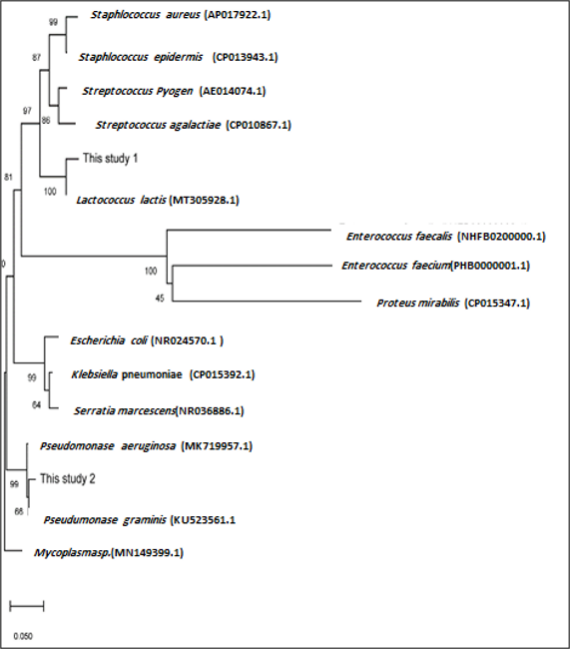
Phylogenetic tree showing the relationship between the nucleotide sequences of the *16S rRNA*gene from different bacteria. *Mycoplasma*sp. *16S rRNA*gene was used as the outgroup to root the tree. The tree was generated using the neighbour-joining method accessed through molecular evolutionary genetic analysis version X software.

*Lactococcus *sp*. *clone UOBVM and* Pseudomonas *sp. clone UOBVM were related to* L. lactis *in the phylogenetic analysis of the *16S rRNA* sequences (MT305928.1).* Pseudomonas* sp. clone UOBVM was more associated with the group of *P. graminis* (KU523561), and this group had common ancestors with the previously reported *P. aeruginosa* (MK719957, Fig. 4). Furthermore, this group was associated with other groups, namely *S. aureus* (APO17922) and *S. epidermidis* (CPO13943), together with the groups of *S. pyogenes* (AEO14074) and *S. agalactiae* (CPO10867).

### Real-time PCR 

**Figure 5.**
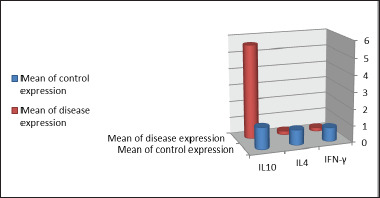
Relative expression of pro-inflammatory cytokines and anti-inflammatory cytokine (IFNγ, IL-4, and IL-10) genes in the milk of cows with clinical mastitis. Using the 2^−∆∆Ct^method, the data are presented as fold changes in gene expression normalized to an endogenous reference gene (GAPDH) and relative to the normal samples (control). Values are represented in mean ± SD and *p*< 0.05.

The expressions of IL-10, IL-4, and IFNγ genes were contrasted in mastitis-free and mastitis-affected animals. Compared with animals without mastitis, IL-10 gene expression was higher in cows with mastitis (*p* < 0.001). Compared to cows infected with clinical mastitis, healthy cows had higher expression of IL-4 and IFNγ genes (*p* < 0.0001; Table 2 and Fig. 5).

## Discussion

The most frequent illness in dairy cows is mastitis, and it causes inflammatory pathology in the udders of the animals and majority of the industry’s financial losses. An essential role in this is the body’s immune system, which supports homeostasis and responds first to all changes in physiological constants.

The innate and adaptive immune systems control MG defense mechanisms, respectively. There are differences in their responsibilities and objectives, yet they are interdependent since they utilize the same route systems. The microbiota and its metabolites play an essential role in maintaining host homeostasis. Antibiotics, infections, or poor nutrition during early development may increase disease susceptibility [19].

**Table 2.** Comparison of the relative gene expressions of IL-10, IL-4, and IFNγ in animals with and without mastitis and the respective significance level of the means.

Variables	Relative gene expression Mean ± SD		
	IL-10	IL-4	IFNγ
Cows with mastitis	5.6 ± 3.510	0.23 ± 0.104	0.22 ± 0.162
Cows without mastitis	1.3 ± 0.772	0.93 ± 0.338	0.84 ± 0.338
Significance level	*p* < 0.001	*p* < 0.0001	*p* < 0.0001

Mastitis is determined by the MG status, innate resistance, and the overall health of the organism. Anatomical, humoral, and cellular characteristics that are both specific and nonspecific combine to form the immune competence of the MG. Phagocytes have a variety of defense mechanisms. These functional phagocytic cells were the polymorph nuclear neutrophil (PMN) leukocyte and the macrophage. Macrophages and epithelial cells work together to start the inflammatory response needed to kill invading germs. They emit chemo-attractants to attract PMN to the infection foci quickly. The creation and release caused the inflammatory reaction to begin [20].

The milk volume was reduced, and the quality of the milk and its products worsened due to an increase in the milk’s concentration of leukocytes and somatic cells. Acute or chronic microbial infections are controlled by resting macrophages, dendritic cells, and other leukocytes after pathogen invasion of the MGs. When bacteria invade the MG mucosa, the body’s natural defenses include an inflammatory reaction. Pathogen-recognizing BME cells, which produce inflammatory mediators, help activate the MG’s inherent immunity. MEC commence downstream signal transduction after MAMPs are recognized by host PRRs, such as TLRs, which lead to the activation of transcription factors. These transcription factors control the expression of genes in MEC, releasing cytokines and chemokines that promote inflammation, type I interferons, and antimicrobial peptides. These inflammatory responses allow pathogens to be removed both locally and systemically [21].

Intramammary inflammation of varying degrees, accompanied by immunological and pathological alterations in the MG tissue, contributes to various physical, chemical, and even microbiological problems in the produced milk.

Traditional bovine mammary (BM) pathogen detection processes take a long time, and most commercial identification systems are not set up to recognize important veterinary diseases like *Escherichia coli*. Diagnostic tests for mastitis should help make a quick, accurate, and conclusive diagnosis of mastitis so that it can be treated. All these tests identify pathogens, changes in milk, body fluids, udder features, or biomarkers/indicators of mastitis.

Molecular approaches are practical tools for developing better diagnostic procedures. Bacterial DNA or RNA can cause diseases; thus, it might serve as a precise target for bacterial DNA amplification and detection [22]. In other studies, depending on the test’s purpose and primer architecture, a variety of DNA-based identification assays can be employed to characterize diseases at distinct evolutionary stages. These methods can be used to find DNA or RNA. Extracting or detecting DNA is more common and often more accessible than removing or detecting RNA because DNA has a better balance than RNA. In contrast to mRNA-based tests, which are much weaker and can only see the best pathogen, DNA-based complete detection tests can detect nonfeasible and inactivated infections. To put it another way, finding antibiotic resistance genes does not mean bacteria are immune to them.

Since its development, the PCR technology has been widely employed in most sectors of biological study to demonstrate the existence of numerous bacteria utilizing PCR based on sequences from the 16S or 23S rDNA sections [23].

Within a few hours, PCR can amplify minute amounts of target DNA. However, the strategy would be impossible to implement if the template was below a particular level. Nested PCR was one of the traditional ways used to overcome this problem. Conventional nested PCR, on the other hand, requires two pairs of primers. The product produced with the inner primers is shorter than the product amplified with the outer primers. For nested PCR, it will be tough for PCR fast-moving target sequences, and it will be easier to employ nested PCR if it is possible to execute it with only one set of primers. First and foremost, we carried out the PCR with only one set of primers for the first time. However, we discovered a strange DNA band on an agarose gel [24].

According to the results obtained after employing universal primers for the *16S rRNA* gene, all clinical mastitis samples included bacterial DNA. These results were corroborated by comparing models from other Internet gene databases with those from the first round of amplification. The detection of bacteria in the environment has been substantially improved by molecular-based approaches such as PCR. PCR-based tests have proven to be more quick, sensitive, specific, and accurate than other methods in routine diagnostics. As a quick method for finding *P. granite *and *Lactococcus lactose*, we turned to nested PCR.

Sequences found in all bacteria have been amplified by utilizing universal lines found in bacteria. The detection of *16S rRNA* in bacteria has been made possible by various primer systems, although each has a different focus on clinical samples and pathogens. Many studies in BM diagnostics with the help of universal 16S bacterial rRNA-based nested PCR methods were published by Joaquim et al. [25] with the help of universal 16S bacterial rRNA-based nested PCR methods.

Real-time PCR offers significant advantages over bacterial cultures and traditional PCR, in addition to those provided by conventional PCR. Because it is faster and more tactile, it is safer for the workers and the environment (no ethidium bromide is used). Also, because it does not require any post-reaction handling (no agarose electrophoresis), RT-mastitis PCR’s pathogen detection sensitivity and specificity are probably 100%. Pathogens can be quantified in infected milk using quantitative RT-PCR; milk cells can be used to extract RNA; complementary DNA can be synthesized; and the expression profile can be quantified using quantitative real-time PCR. For gene expression, the Ct was calculated by comparing the target gene’s expression to the housekeeping gene’s expression [26]. In a turn of events, the PCR procedure has been generally utilized in many areas of scientific research. In a couple of hours, PCR can intensify minute measures of target DNA. The elimination of culture, rapidity, and ease of analysis are the main benefits of PCR. The current study relied on PCR amplification of the DNA region coding for rRNA. Several essential areas exist for the construction of universal probes due to the availability of hypervariable regions, which enable the production of highly specific oligonucleotide probes. Furthermore, because rDNA is present in several copies, signal augmentation is possible. PCR has excellent specificity and sensitivity for accurate diagnosis, making it one of the most widely accepted and widely utilized diagnostic techniques. In diagnosing a condition with a wide range of symptoms and clinical presentations, PCR can help quickly and accurately diagnose [27].

Two separate PCRs are used to complete the procedure. First, primers covering both ends of the target sequence are used in the reaction, and some different sequences flank the target sequence on each side. After the initial response, primers that bind to the target sequence and are located within the first PCR’s amplified sequence are used in a second reaction to the first PCR’s results. The use of nested PCR minimizes DNA template nonspecific amplification. It minimizes nonspecific binding since most of the amplicons from the first reaction include only the target sequence and its surrounding sequences in the second reaction.

The *16S rRNA* or rDNA sequences examination has grown important in studying bacterial relationships and is now routinely utilized for bacterial identification. The phylogenetic tree identifies the genus and closest relatives of the query DNA strain by comparing them to other sequences in the database. The creation of new platforms for genotypic, chemical taxonomy, and phenotypic research. A separate taxon than the one being researched must be used as an outgroup when constructing a phylogenetic tree showing the strains’ taxonomic tree of origin [28].

Sequence alignment is used in this study to arrange DNA, RNA, or protein sequences to find regions of similarity caused by functional, structural, or evolutionary links between the sequences. It is common to display aligned nucleotide or amino acid sequences as rows in a matrix. Identical or similar characteristics are aligned in subsequent columns by inserting gaps between the residues. It is also used to calculate the cost of the distance between strings in plain language or in financial data using sequence alignments that are not biological.

Both phylogenetics and sequence alignment necessitates evaluating sequence relatedness and proving evolutionary relationships between different biological species or entities based on physical or genetic similarities and differences. Hence, the two fields are intertwined. To categorize the evolutionary relationships between homologous genes found in the genomes of different species, the science of phylogenetics relies heavily on sequence alignments to build and analyze phylogenetic trees. The evolutionary distance between sequences in a query set is qualitatively related to the degree to which they differ [29].

Accordingly, the outer and inner UNI primers of two 16S bacterial rRNAs were used in the current study. After the first and second nested PCR runs, amplicons of the predicted sizes of 709 and 287-bp were observed in 70 (46.7%) cases, which is in line with *S. aureus*, *S. pyogenes*,* S. epidermidis*, *S. agalactiae*, *Enterococcus faecium*, *E. faecalis*, *K. pneumoniae*, *Proteus mirabilis*, *E. coli*,* P. aeruginosa*, and *S. marcescens*. Many authors developed molecular probes that react in PCR with bacterial DNA from bovine milk, allowing for direct and rapid detection of *E. coli*,* S. aureus*,* S. agalactiae*,* S. dysgalactiae*, *S. parauberis*, and* S. uberis*. Furthermore, two universal primer sets were created to respond similarly [30]. After the first and second runs, amplicons of the predicted sizes of 709 and 287-bp were detected. Mastitis is a severe, multifaceted, and genetically and environmentally influenced infection. It is the most common and expensive infection in cows [1]. Different microorganisms are linked to mastitis in other geographic locations based on the differences in the management strategies used in various countries. The environmental bacterial reference may be made for mastitis in this study. *Lactococcus* sp. clone UOBVM and *Pseudomonas* sp. clone UOBVM were deposited in the GenBank database under accession numbers MW020033 and MW020096, respectively. The two clones were related to environmental events, one of which (*Pseudomonas* sp. clone UOBVM). However, the association of the other clone (*Lactococcus* sp. clone UOBVM) with mastitis has been reported in other studies [7].

The high-resolution power of sequencing in identification compared with culture-based approaches, unhygienic milking techniques, poor housing conditions, and/or overuse of antibiotics could all be factors in the existence of environmental bacteria as a cause of mastitis.

This study looks at expressions of genes, such as IL-4, IL-10, and IFNγ, linked to immune response mechanisms in mastitis. Cows with mastitis had higher levels of IL-10 gene expression than healthy animals in the sample. On the contrary, the expression of other genes was lower in mastitis-affected cows than healthy ones; the difference between the two groups was significant. 

To control the immune response, cytokines work in conjunction with cytokine inhibitors and soluble cytokine receptors. Inhibition of natural killer cells, macrophages, and Th1 immune cell production of IL-1 and tumor necrosis factor by the anti-inflammatory cytokine IL-10. More and more research demonstrates their physiological significance in inflammation and their pathologic role in systemic inflammatory disorders. Anti-inflammatory cytokines such as IL-1 Ra, IL-4, IL-6, IL-10, IL-11, and IL-13 [31]. 

Cell differentiation can be influenced by IL-4, a potent pleiotropic cytokine. The human immune system’s most essential anti-inflammatory cytokine is IL-10. Th1 cytokines like IL-2 and IFN are effectively inhibited by it. The cytokine IL-4 inhibits IFNγ’s development. Its main function is to regulate immunoglobulin E-mediated immune function. IL-4 also promotes the production of T helper 0 and T helper 2 cells, resulting in a humoral immune response. IFN’s biological effects are simple to explain because of its capacity to activate or inhibit the production of specific target genes. 

All IFN’s biological effects are dependent on IFN’s binding to certain cell surface receptors, which are found on nearly all cell types. As a result, elevated amounts of IL-10 may have slowed IFN production. The new findings are in line with previous research that found that. In cows with mastitis, the expressions of IL-4 and IFNγ genes were significantly lower than those in healthy animals (*p *< 0.0001).

Further research is needed to best represent and comprehend mastitis-affected cows’ gene expression profiles, given that no Iraqi studies of this kind are currently available. Thus, the findings of this study cannot be compared with those from other countries. The current cytokine expression findings indicate that an immune response can be caused depending on the bacterial strain and host, with large individual variance observed. In line with the current findings, an *in vitro* analysis was conducted. The MG epithelial cells respond differently to different *S. aureus* strains. In addition, the level and degree of expression of the genes investigated (e.g., IL-10) changed according to infection stage (3, 10, and 24 h after the addition of bacteria to the cell culture). These numerous responses indicate a shift in behavior.

The milk samples in this study were collected as soon as possible because telling when the animal is infected is difficult as clinical symptoms of mastitis emerge on various days even though they are infected on the same day. In addition, no one knew what strain of bacteria was responsible for the infection, and it may have been fatal in some situations. Mammitis is another condition that has many causes, many of which are genetic. All the genes tested were significantly suppressed in each of the animals (*p ˂ *0.001). Thus, they are good indicators of mastitis in the characters tested.

Finally, Gram-positive and Gram-negative bacteria were linked to mastitis. The IL-4. IL-10, and IFNγ genes were differentially expressed in mastitis and healthy conditions (*p* ˂ 0.01), indicating that these genes are strong indicators of mastitis in the conditions studied. Additional studies would be conducted to explore other genes.

## Conclusion

This study aims to identify the bacteria that caused mastitis either by Gram-positive or Gram-negative bacteria. Bacterial DNA or RNA, in addition to being critical for disease transmission, could also serve as a precise target for bacterial amplification. Nested PCR used for the DNA isolated from the somatic cell of cow milk mastitis was predicted to be multiplied by uniform bacterial sections of the *16S rRNA* gene. Mastitis caused by Gram-positive and Gram-negative bacteria was used to assess the expressions of IL-4, IL-10, and IFNγ genetic features connected to the immune system response pathways to mastitis. Real-time PCR makes mRNA expression measurements easier, faster, and more sensitive. It may be exceedingly precise and dependable with proper tuning, making it an ideal standard approach for cytokine mRNA measurement.

## List of Abbreviations

BM, bovine mammary; BME, bovine mammary epithelial; cDNA, complemented deoxyribonucleic acid; DNA, deoxyribonucleic acid; GAPDH, glyceraldehyde-3-phosphate dehydrogenase; MG, mammary gland; MEC, mammary epithelial cells; NCBI, National Center for Biotechnology Information; PCR, polymerase chain reaction; PMN, polymorph nuclear neutrophil; rDNA, ribosomal deoxyribonucleic acid; RNA, ribonucleic acid; *16S rRNA*, 16S ribosomal ribonucleic acid; SCC, somatic cell count; TNF, tumor necrosis factor.
